# Advancing nitrogen diagnostics in plants through bioimpedance spectroscopy: current evidence and future perspectives—a review

**DOI:** 10.1007/s00425-026-04934-y

**Published:** 2026-02-02

**Authors:** Flórián Kovács, Ákos Odry, Zoltán Vizvári, Ingrid Melinda Gyalai, Adrienn Szarvas, Gideon Adu Donyina, Péter Odry, Katalin Juhos

**Affiliations:** 1 Department of Agro-Environmental Studies, Hungarian University of Agriculture and Life Sciences, Villányi Str. 29-43, 1118 Budapest, Hungary; 2 Faculty of Agriculture, Institute of Plant Sciences and Environmental Protection, University of Szeged, 6800 Hódmezővásárhely, Hungary; 3 Department of Mechatronics and Automation, Faculty of Engineering, University of Szeged, Moszkvai Krt. 9, 6725 Szeged, Hungary; 4 Department of Control Engineering and Information Technology, University of Dunaújváros, Táncsics Mihály U. 1, 2400 Dunaújváros, Hungary; 5 John Von Neumann Faculty of Informatics, Biomatics and Applied Artificial Intelligence Institute, Óbuda University, Bécsi Str. 96, Budapest, 1034 Hungary; 6 Bioimpedance Technologies Research Center, University Research and Innovation Center, Óbuda University, Bécsi Str. 96, Budapest, 1034 Hungary; 7 Multidisciplinary Medical and Engineering Cellular Bioimpedance Research Group, Szentagothai Research Centre, University of Pecs, Ifjusag Str. 20, 7624 Pecs, Hungary

**Keywords:** Bioimpedance spectroscopy, Nitrogen deficiency, Equivalent circuit model, Non-destructive diagnostics

## Abstract

Nitrogen (N) is an essential macronutrient that plays a central role in photosynthesis, metabolism, and crop productivity. Accurate and non-destructive evaluation of plant N status is essential for improving N use efficiency and sustainable fertilization. Bioimpedance spectroscopy (BIS) has emerged as a promising tool for in vivo assessment of plant physiological state; however, its application to nutrient monitoring remains limited. Previous studies show that N deficiency significantly alters extracellular and intracellular fluid resistances and reduces cell membrane capacitance, reflecting impaired ion conductivity, loss of membrane integrity, and changes in vacuole storage. These alterations can be detected in vivo within specific frequency ranges and often correlate with leaf N content, but most studies considered only total N and did not account for inorganic nitrate (NO_3_⁻) forms or water-related effects. Future research should combine BIS with direct apoplastic NO_3_⁻ measurements and factorial N and water experiments to distinguish nutrient-specific responses from drought-induced changes. Applying advanced equivalent circuit models, such as the Double-Shell (DBS) model, could strengthen physiological interpretation and associate impedance parameters with cellular functions. Addressing these issues will enable BIS to become a reliable, non-destructive diagnostic method for N monitoring.

## Introduction

Bioimpedance spectroscopy (BIS) is a non-invasive technique that measures the electrical properties of biological tissues, providing insights into the physiological state of plants, including water content (Zhou et al. 2024) and cellular integrity. Current research on BIS in crops focuses primarily on plant water status and general health (Jamaludin et al. 2015; Wang et al. 2020; Garlando et al. 2022; Zhang et al. 2022a, 2024; Reynolds et al. 2023; Serrano-Finetti et al. 2023), with limited research on nutrient-specific effects.

While a wide range of destructive and non-destructive techniques exists for assessing plant nutrient status, the present Introduction focuses exclusively on BIS to highlight its unique advantages as a non-invasive, in vivo approach; a comparative overview of alternative methods is therefore addressed separately in Sect. "Advances in N monitoring: from destructive method to in vivo sensing".

However, several studies have demonstrated the potential of BIS to detect nutrient deficiencies such as nitrogen (N) (Muñoz-Huerta et al. 2014; Li et al. 2017; Basak et al. 2020; Kovács et al. 2025b, d, a), phosphorus (P) (Meiqing et al. 2016), potassium (K) (Jinyang et al. 2016), and iron (Fe) (Hamed et al. 2023) deficiencies. Nevertheless, studies focusing specifically on nutrient uptake dynamics and fertilization management remain extremely limited, highlighting the need for more systematic investigations into the relationship between BIS parameters and nutrient supply.

At the cellular level, BIS signals originate from how different components of plant tissues conduct or store electric charge (Prasad and Roy 2020). The intercellular fluid space mainly affects the low-frequency response, which is described by the extracellular fluid resistance, whereas the intracellular compartment dominates the higher-frequency range through the intracellular fluid resistance. The cell membrane separates these two regions and acts as a thin insulating layer that limits ion movement, represented by the cell membrane capacitance. More advanced equivalent circuit models also include the vacuole and its surrounding membrane, which are described by the vacuole fluid resistance and vacuole membrane capacitance (Zhang et al. 1992). These electrical parameters differ with the plant’s nutrient status and allow the measured impedance values to be related to the physiological condition of the tissues (Kovács et al. 2025d).

In the absence of N, the resistance of the extracellular and intracellular fluids in the leaf, as well as the capacitance of the cell membrane, changes significantly (Li et al. 2017; Kovács et al. 2025d). This interpretation is complicated by the fact that most studies have relied only on the Kjeldahl method to determine total N content and compared it with BIS parameters (Muñoz-Huerta et al. 2014; Li et al. 2017; Basak et al. 2020). Furthermore, these studies did not detail the relationship between leaf extracellular fluid resistance and the concentrations of nitrate (NO_3_⁻) or ammonium (NH_4_⁺) nor did they discuss their physiological relevance (Muñoz-Huerta et al. 2014; Li et al. 2017; Basak et al. 2020). Earlier BIS studies therefore failed to clarify how ionic concentrations influence the electrical resistance of the extracellular and intracellular compartments in leaves. In contrast, two recent investigations (Kovács et al. 2025b, d) have demonstrated a clear correlation between NO_3_⁻ concentration and extracellular fluid resistance, suggesting that BIS parameters can sensitively reflect changes in NO_3_⁻ availability within the leaf tissue.

While the relationship between BIS signals and plant water deficit is well established (Jamaludin et al. 2015; Wang et al. 2020; Garlando et al. 2022; Zhang et al. 2022a, 2024; Reynolds et al. 2023; Serrano-Finetti et al. 2023), the use of BIS for N monitoring in plants remains challenging due to the saturation of leaf NO_3_⁻ in the extracellular fluid (Crawford and Glass 1998; Nikolic et al. 2012; Hu et al. 2014) and the bidirectional exchange (efflux/influx) of NO_3_⁻ ions between the extracellular and intracellular spaces (Crawford and Glass 1998). This challenge is further complicated by the fact that water deficit also significantly alters the resistance of both compartments, making it difficult to distinguish whether changes in extracellular fluid resistance are caused by water deficit or N deficiency (Kovács et al. 2025b, d).

Therefore, more advanced tissue- and cell-specific N assay strategies are needed in the future to enable the effective use of BIS for N monitoring. Once these limitations are addressed, BIS could serve as a powerful complementary approach to NO_3_⁻ -selective electrodes (Ibrahim et al. 2022) for assessing N status in plants. In this context, the present study aims to investigate how BIS parameters respond to different levels of nitrogen supply, providing new insights into the electrical mechanisms underlying N-related physiological changes in plants.

## Aim and scope of the review

Recent advances in BIS have demonstrated its high sensitivity to physiological changes in plants under water deficit and other abiotic stresses. However, its application to nutrient-related processes—particularly N uptake, transport, and assimilation—remains insufficiently explored and lacks conceptual integration.

This review summarizes the current knowledge on how BIS parameters relate to plant N status and explains how electrical models can describe N-related changes at the tissue and cell levels. It also points out the main methodological gaps, such as the lack of standardized circuit models, differences in electrode setup, and the few studies that compare BIS results with physiological or chemical indicators of N status.

Finally, this review highlights future directions for improving BIS as a tool for monitoring nutrients. Understanding the relationship between the electrical behavior of plant tissues and N metabolism may help make BIS a reliable, non-destructive, and accurate method for assessing nutrient status in living plants.

Sects. "The role of N in plant growth and metabolism" and "Advances in N monitoring: from destructive method to in vivo sensing" summarize the physiological background of N uptake, assimilation, and monitoring in plants, highlighting the key processes that determine N availability and signaling. Sects. "Principles and applications of bioimpedance spectroscopy in plant physiological research" and "Equivalent circuit models of bioimpedance and their physiological interpretation" describe the physical principles of BIS and the main equivalent circuit models used to interpret plant electrical responses, emphasizing their physiological relevance. Sect. "Limitations and challenges of bioimpedance applications in nutrient uptake and N status assessment in plants" focuses on the relationship between BIS parameters and N status, presenting recent findings on how BIS characteristics reflect N-related changes in plant tissues.

## The role of N in plant growth and metabolism

N is one of the most essential macronutrients for plants, playing a fundamental role in metabolism and biomass production, as it is a key component of amino acids and proteins and is required for chlorophyll biosynthesis as well as for nucleic acids, membranes, and cell walls (Foyer and Zhang 2010; Mu and Chen 2021).

Plants absorb N in both inorganic and organic forms (Muratore et al. 2021); however, under most soil conditions uptake occurs predominantly as ammonium (NH_4_⁺) and nitrate (NO_3_⁻). Among these, NO_3_⁻ is the most abundant and widely absorbed inorganic form in soils, particularly under aerobic conditions (O’Brien et al. 2016). In contrast, NH_4_⁺ becomes dominant in more acidic soils and anaerobic environments. Both forms exert distinct effects on plant metabolism and cellular pH regulation, and therefore the physiological responses of plants depend strongly on the specific N source supplied (O’Brien et al. 2016; Li et al. 2024).

N uptake in plants is an energy-demanding process that depends on the morphological characteristics of the root system, the availability of different N forms in the soil, and various environmental factors (Li et al. 2013; Hu et al. 2014). In addition to NO_3_⁻ and NH_4_⁺, plants can also utilize small amounts of organic N sources, such as amino acids and amides (Lea and Miflin 2010; Li et al. 2013; O’Brien et al. 2016; Rashid et al. 2022).

The uptake and metabolism of different N forms not only influence nutrient status but also fundamentally determine the plant’s physiological responses and stress tolerance (Britto and Kronzucker 2002; Li et al. 2022, 2024). These processes are regulated by specific transporter families (Morales de Los Ríos et al. 2025) that control N uptake and intracellular distribution. Moreover, the rhizosphere microbiome and mycorrhizal symbioses significantly affect the availability and transformation of N forms in the soil, thereby influencing overall N uptake and utilization (Tegeder and Masclaux-Daubresse 2018).

NO_3_⁻ uptake in plants can involve proton-coupled transport systems, and many transporters implicated in NO_3_⁻ nutrition belong to the NRT1/PTR FAMILY (NPF) (Morales de Los Ríos et al. 2025). Within this family, AtNPF6.3 (formerly AtNRT1.1/CHL1) is a central and extensively studied member of the NPF6 clade. It has been reported to mediate NO_3_⁻ transport over a broad external concentration range, and a phosphorylation-based regulation at T101 (via CIPK23) has been proposed to mediate an apparent switch between affinity modes. However, this affinity shift remains controversial, as T101 phosphorylation does not alter the NO_3_⁻ binding affinity (KD), and the occurrence of high-affinity behavior is strongly dependent on the experimental setup. Moreover, the reported dual-affinity behavior can result from fitting uptake rates with two separate Michaelis–Menten equations, whereas a single Michaelis–Menten model has been shown to provide the best fit to published datasets. Beyond transport, AtNPF6.3 has a well-established signaling function and is therefore often described as a NO_3_⁻ transceptor (Ho et al. 2009; Morales de Los Ríos et al. 2025).

Once NO_3_⁻ enters the root, part of it is assimilated in the root, while another portion is transported to the shoot via the xylem. NO_3_⁻ assimilation is a compartmentalized process: the reduction of NO_3_⁻ to NO_2_⁻ by nitrate reductase (NR) occurs in the cytosol, whereas the subsequent reduction of NO_2_⁻ to NH_4_⁺ by nitrite reductase (NiR) takes place in the plastids. The resulting NH_4_⁺ is then incorporated into amino acids via the GS/GOGAT cycle, which can be localized entirely in plastids or distributed between the plastids and the cytosol. Transporters involved in xylem NO_3_⁻ loading and unloading play key roles in regulating root-to-shoot NO_3_⁻ translocation, mediating both the efflux of NO_3_⁻ into the xylem and its retrieval back into the root (Nikolic et al. 2012; Morales de Los Ríos et al. 2025).

After reaching the leaves, NO_3_⁻ first enters the apoplast, from which its uptake into the symplast is driven by the proton motive force generated by the plasma membrane H⁺–ATPase. Nikolic et al. (2012) demonstrated in cucumber leaf disks that this inducible, saturable leaf NO_3_⁻ uptake is light-dependent and pH-sensitive (optimal at pH 5.5–6.5), and that inhibition of H⁺–ATPase activity drastically reduces NO_3_⁻ absorption. During the early phase of induction, the NO_3_⁻ concentration in the apoplast increases rapidly but then declines as symplastic uptake increases, indicating that the apoplast acts as a buffer and distribution compartment in leaf N supply.

NO_3_⁻ assimilation is closely related to photosynthesis, since the reduction steps require both energy and reducing agents, as well as carbon skeletons into which N is incorporated. The activity of NR follows a daily cycle, being higher during the day and lower at night. This variation is associated with the light-dependent redox state and the flow of carbon compounds generated through photosynthesis (Nikolic et al. 2012).

In roots, the assimilated NO_3_⁻ is mainly converted into amino acids (glutamine and asparagine), which are then transported through the phloem to young shoots and developing organs (Tegeder and Masclaux-Daubresse 2018). In leaves, a large part of NO_3_⁻ is stored in the vacuoles (Crawford and Glass 1998). This storage pool also has osmoregulatory and buffering functions: during the day, NR and NiR activity depletes the vacuoles’ stores, while at night they are refilled.

During senescence, N from leaf proteins is remobilized and transported to the seeds or young tissues. In the tonoplast, the AtCLCa NO_3_⁻ /H⁺ antiporter mediates vacuole storage, which stabilizes the cytosolic NO_3_⁻ level and the cell pH (Tegeder and Masclaux-Daubresse 2018).

The movement of NO_3_⁻ inside the leaf occurs through three coordinated processes:

i) in the extracellular fluid space,
ii) in the intracellular fluid space (uptake driven by H⁺–ATPase through NO_3_⁻ transporters, including members of the NPF family, and assimilation via the NR/NiR pathway), and
iii) in the vacuole (NO_3_⁻ storage and osmoregulation).

According to Nikolic et al. (2012), the peak in extracellular NO_3_⁻ concentration comes before the maximum of intracellular uptake and H⁺–ATPase activation, suggesting that the extracellular acts as a short-term “buffer” that helps balance NO_3_⁻ levels under changing light, pH, and transpiration conditions.

The uptake of NH_4_⁺ mainly occurs through the AMT1 and AMT2 transporter proteins, which form a high-affinity system (Yuan et al. 2007). Although the direct incorporation of NH_4_⁺ into cells is more energy-efficient than NO_3_⁻ assimilation, the accumulation of NH_4_⁺ inside the cell can be toxic because it disturbs pH and ion balance (Britto and Kronzucker 2002). To avoid this, NH_4_⁺ is rapidly incorporated into organic compounds, mainly through the glutamine synthetase/glutamate synthase (GS/GOGAT) cycle (Lea and Azevedo 2006). Low-affinity NH_4_⁺ transport can also occur through aquaporins and non-selective cation channels. The activity of AMT transporters can be quickly turned off by phosphorylation (e.g., by CIPK23), and it is closely linked to K^+^ homeostasis and the acidification of the apoplast (Tegeder and Masclaux-Daubresse 2018).

In seed plants, glutamine synthetase (GSIIe) is represented by three phylogenetic groups (GS1a, GS1b, and plastidic GS2). GS2 predominates in photosynthetic tissues (e.g., leaf mesophyll), where it reassimilates NH_4_⁺ from NO_3_⁻ reduction and photorespiration, whereas cytosolic GS1 isoenzymes are present across organs and contribute to primary N assimilation (notably in roots) as well as to N remobilization and translocation in shoots; GS1b is typically a small multigene family with organ-dependent roles, and GS1a (present in gymnosperms and some basal angiosperms/Magnoliidae) may overlap functionally with GS2 in photosynthetic cells (Valderrama-Martín et al. 2022).

The ratio between NO_3_⁻ and NH_4_⁺ strongly determines the N use efficiency (NUE) of plants. A balanced NO_3_⁻: NH_4_⁺ ratio supports cellular pH homeostasis, optimizes photosynthesis and ion balance, while excessive NH_4_⁺ supply can lead to acidification, osmotic stress, and membrane damage (Hachiya and Sakakibara 2017). A moderate proportion of ammonium (20–40% of total N) is generally beneficial for many leafy vegetables, whereas a high (> 40–50%) NH_4_⁺ ratio often causes toxicity symptoms and yield reduction.

Overall, the dynamic balance between NO_3_⁻ and NH_4_⁺ forms, together with the coordinated activity of transporters, enzymes, and signaling networks, determines the nitrogen use efficiency of plants.

## Advances in N monitoring: from destructive method to in vivo sensing

Determining the N status of plants is a central issue in both plant physiology and agronomy, as N is the most abundant essential macronutrient that fundamentally influences photosynthetic activity, metabolism, and yield potential (Muñoz-Huerta et al. 2014).

Leaf N content is closely related to chlorophyll concentration, photosynthetic rate, and total biomass (Zotarelli et al. 2007; Muñoz-Huerta et al. 2014; Gao et al. 2023; Lal et al. 2023). Accurate and timely determination of plant N status is essential for improving nitrogen use efficiency (NUE) and reducing environmental impacts caused by over-fertilization. NUE represents the ratio of absorbed and assimilated nitrogen and plays a key role in sustainable agriculture (Berger et al. 2020).

Several methods have been developed to estimate plant N content, which can be broadly categorized into direct (destructive) and indirect (non-destructive) approaches (Zotarelli et al. 2007; Muñoz-Huerta et al. 2014).

In recent years, the integration of satellite- and UAV-based SIF (solar-induced chlorophyll fluorescence) data, which enables direct monitoring of photosynthetic activity, has significantly improved the spatial and temporal sensitivity of N diagnostics. This technique is particularly effective when combined with vegetation indices such as the NDRE (Normalized Difference Red Edge Index) or NBI (Nitrogen Balance Index), together with proper field calibration (Padilla et al. 2016; Hou et al. 2023).

Analytical N determination methods measure the actual N concentration in plant tissues using chemical or physical techniques (Muñoz-Huerta et al. 2014). The most widely used procedure is the Kjeldahl method, in which organic N is digested into NH_4_⁺ ions under acidic conditions and then quantified by titration (Nelson and Sommers 1980). This method provides high accuracy and reproducibility; however, it is time-consuming, labor-intensive, requires hazardous chemicals, and does not directly measure NO_3_⁻ or NO_2_⁻ forms, thus only the total N content can be determined.

The Dumas method is based on combustion, during which N in the sample is converted into N gas (N_2_) and its concentration is then measured using gas analysis. This method is faster and can be fully automated; however, its high equipment cost and the need for laboratory sample preparation limit its applicability for field use (Nelson and Sommers 1980; Chang and Robison 2003; Saint-Denis and Goupy 2004; Padilla et al. 2016).

In recent decades, several spectroscopic techniques have emerged, such as near-infrared spectroscopy (NIR), which analyzes the absorption spectrum of plant samples and estimates N content based on correlation models derived from the spectral data (Liang et al. 2018). Additionally, Fourier-transform infrared spectroscopy (FTIR) and Raman spectroscopy have gained importance, as they allow a more detailed analysis of nitrogen-containing compounds in plant tissues, particularly in heterogeneous samples (Losacco et al. 2022; Padilla et al. 2018a, b).

The main advantages of NIR measurements are their speed and minimal sample preparation requirements; however, the accuracy of this method depends strongly on the quality of calibration curves and on factors such as plant species, water content, and cell structure variability (Liang et al. 2018). In the NIR range, absorption bands associated with proteins and N (approximately 1510–1520, 1690, 1940, and 2060–2350 nm) often overlap with those of other components such as water, lignin, cellulose, and starch. It is important to note that NIR spectra generally include information about the same chemical components, which are distributed across multiple wavelength ranges due to overtones and combination vibrations. As a result, the separation of signals associated with N from other matrix components is often performed using spectral preprocessing, deconvolution, and multivariate machine learning methods, similar to the analysis of other mixed spectral signals, rather than relying on a single absorption band (Azadnia et al. 2023). Therefore, proper spectral separation and control of water content are critical for reliable N estimation (Berger et al. 2020). Although these methods are accurate, their application is limited by cost and sample destruction, which has led to a focus on non-destructive techniques (Zotarelli et al. 2007; Ji-Yong et al. 2012; Xiao et al. 2022).

In recent years, laser-induced breakdown spectroscopy (LIBS) has also gained increasing attention for N determination in plant samples. The technique has gradually advanced from laboratory settings toward field applications: using single-shot measurements, LIBS has been demonstrated to provide in situ, practically non-destructive estimation of N content in leaf samples (e.g., fresh lettuce). When combined with machine learning algorithms, this approach achieved high accuracy (Hossen et al. 2021).

Among the most widely used approaches for rapid and non-destructive determination of plant N content are optical methods, which rely on the reflectance, transmittance, and fluorescence properties of leaves or plant canopies (Homolová et al. 2013; Gholizadeh et al. 2017). A common feature of these techniques is that they do not measure N directly; instead, they assess parameters that correlate with chlorophyll content, since chlorophyll molecules contain a substantial amount of N (Riccardi et al. 2014; Berger et al. 2020).

One of the most commonly used portable instruments is the SPAD (Soil and Plant Analysis Development) chlorophyll meter, which measures the green color intensity (absorbance) of leaves at two wavelengths, typically between 650 and 940 nm. The obtained values are empirically correlated with leaf nitrogen content (Padilla et al. 2018a, b; Padilla et al. 2016). The SPAD meter is fast and non-invasive; however, its readings can be influenced by several factors such as leaf thickness, species-specific optical properties, soil moisture, and light intensity (Xiong et al. 2015). Moreover, the method shows a non-linear relationship with the actual leaf N content, which means that results must be calibrated for each species and developmental stage (Berger et al. 2020).

Remote sensing techniques, such as hyperspectral imaging and the NDVI (Normalized Difference Vegetation Index), are also widely applied non-destructive methods for estimating plant N status (Gitelson et al. 2002; Padilla et al. 2018a, b). Hyperspectral measurements record the reflectance properties of leaves or canopies across hundreds of narrow spectral bands, enabling more precise detection of spectral features related to nitrogen and chlorophyll content (Cabrera-Bosquet et al. 2009; Luo et al. 2016; Liang et al. 2018). NDVI, calculated from the ratio of near-infrared to red wavelengths, serves as an indirect indicator of vegetation cover and photosynthetic activity (Ramoelo and Cho 2018). Various spectral indices, such as parameters derived from the N-PROSAIL model, allow mapping of canopy-level nitrogen distribution (Gitelson et al. 2002; Padilla et al. 2018a, b).

The main limitations of hyperspectral systems include their high cost and complex data management requirements: they demand highly sensitive detectors, fast computational power, and large data storage, while environmental factors (soil background, light conditions, canopy structure, and vegetation type) can distort nitrogen estimation (Berger et al. 2020). Nevertheless, UAV and satellite platforms equipped with multispectral or hyperspectral sensors play an increasingly important role in supporting site-specific nitrogen management in precision agriculture. UAV-based estimation of canopy-level biomass and nitrogen content has already shown promising applicability in several crops (Cabrera-Bosquet et al. 2009; Luo et al. 2016; Liang et al. 2018).

Other modern approaches include RGB-based image analysis and machine learning techniques, which estimate N status by exploiting the relationship between leaf color (e.g., Dark Green Color Index, DGCI) and chlorophyll content (Riccardi et al. 2014). Recently, multispectral structured light imaging methods have been introduced to monitor nutrient status dynamics through changes in leaf surface structures (Nguyen et al. 2015). Algorithms based on artificial neural networks (ANN) and regression models (Kaul et al. 2005) can improve estimation accuracy; however, environmental factors such as light conditions, air humidity, and soil reflectance can strongly affect the results (Sun et al. 2017).

The main advantages of the above optical methods are their speed, non-destructiveness, and suitability for field applications. However, their common limitations include high sensitivity to environmental conditions, the need for species-specific calibration, and the non-linear relationship with actual nitrogen content (Muñoz-Huerta et al. 2014; Berger et al. 2020). In recent years, portable multispectral devices have been developed that can directly estimate leaf N concentration under field conditions using species- and crop-specific models. These instruments represent an intermediate approach between traditional SPAD/NBI measurements and more complex hyperspectral systems (Jiang et al. 2021; Karaca et al. 2023; Yu et al. 2024).

SIF (solar-induced chlorophyll fluorescence)-based methods, which are directly linked to photosynthesis, can sensitively indicate plant N status even at the canopy level. High-resolution satellite data enable fine-scale monitoring, particularly under stress conditions such as drought. The accuracy of hyperspectral remote sensing can be further improved by applying machine learning algorithms (e.g., RF, XGBoost), especially when incorporating Sentinel-2 red-edge bands into the modeling process (Yin et al. 2023; Xu et al. 2024).

As summarized in Table 1, existing methods for plant N determination differ substantially in their level of destructiveness, specificity, accuracy, and field applicability. Reference analytical techniques provide reliable quantification of total N content but are inherently destructive and therefore unsuitable for repeated or in situ measurements. Optical and spectral approaches allow rapid, non-destructive assessment in the field; however, they are based on indirect indicators of N status and require extensive calibration across species and environmental conditions.

**Table 1:** Comparison of destructive and non-destructive methods for plant N determination

Method	Destructive?	Specificity to N forms	Sensitivity / Accuracy	Field applicability	Measurement speed	Cost	Sample preparation	Calibration Requirement	Environmental sensitivity	References
Kjeldahl Method	Yes	Total N element content	High; laboratory reference method	No	Slow	Medium (chemicals and labor)	Drying, grinding, acid digestion	No	Low (lab-controlled)	Domini et al. (2009), Sáez-Plaza et al. (2013)
Dumas Method	Yes	Total N element content	High; fast and automatable	No	Fast	High (instrument acquisition)	Drying and grinding	No	Very low (fully controlled combustion)	Etheridge et al. (1998), Jung et al. (2003)
NIR Spectroscopy	Protocol-dependent*	Indirect (calibrated total N estimation)	Moderate-high (model- and calibration-dependent)	Limited (portable versions exist)	Fast	High	Variable (fresh or dried, depending on calibration)	Yes (species-specific)	High (water, matrix, species)	Xiao et al. (2022), Bao et al. (2024)
FTIR Spectroscopy	Protocol-dependent*	Amide I-II bands; indirect protein-N estimation	Moderate-good; water-sensitive	Limited (mostly lab)	Fast	High	Grinding (KBr pellet) or ATR (minimal / none)	Yes	High	Wu et al. (2019)
Raman Spectroscopy	Non-destructive (power-dependent)	Molecular specificity (N-related bands)	High spatial and molecular specificity; SNR-limited	Emerging (portable systems)	Fast	High	Minimal	Yes (sample- and matrix-dependent)	Moderate	Hou et al. (2025), Tuccio et al. (2025)
LIBS (Laser-Induced Breakdown Spectroscopy)	Minimally destructive	Elemental N (primarily via CN emission; no molecular speciation)	Moderate to good; improved with ML and multivariate analysis	Yes (portable)	Fast	High	Minimal	Yes (model dependent)	Moderate	Lu et al. (2024)
SPAD meter	No	Indirect (chlorophyll-N correlation)	Low-moderate (saturates at high N/chlorophyll)	Yes (handheld)	Fast	Low	None	Yes (species/stage)	High	Padilla et al. (2020), Karaca et al. (2023)
NDVI (Normalized difference vegetation index)	No	Indirect (canopy-level proxy)	Moderate; saturates at high biomass	Yes (remote sensing)	Fast	Low	None	Yes (site-specific)	High	Raun et al. (2002), Hossen et al. (2021), Jiang et al. (2021)
Hyperspectral Imaging	No	Indirect (spectral indices related to N-linked pigments and proteins)	Very high (with multivariate/ML models)	Yes (UAV/satellite)	Fast (processing-intensive)	High	None	Yes	High	Cohen et al. (2010), Hu et al. (2025)
SIF (Solar-Induced Fluorescence)	No	Indirect (photosynthesis-coupled fluorescence; proxy for N status)	High; improves with indices	Yes (UAV/satellite)	Fast / repeatable	High	None	Yes	High	Jia et al. (2018), Li et al. (2025)
RGB + Machine Learning	No	Indirect (image-based chlorophyll estimation)	Highly variable; strongly model- and dataset-dependent	Yes (camera/phone/UAV)	Fast	Low	None	Yes (training-dependent)	High	Ge et al. (2025), Martins et al. (2025)
Ion-Selective Electrodes (ISE)	No	NO_3_⁻ activity (not total concentration)	Moderate; needs stabilization	High (portable, in situ)	Fast	Medium (low for sensor, higher for system)	Minimal	Yes	High (temperature, ionic strength, fouling)	Ibrahim et al. (2022)

**Methodological note**: destructiveness is method- and protocol-dependent. Methods marked with * are non-destructive in principle but are often rendered destructive by laboratory sample preparation (e.g., drying and grinding). Portable or in situ implementations, however, enable non-destructive measurements. Sample preparation requirements also vary; methods marked with ** typically involve drying and grinding under conventional protocols, whereas ATR-FTIR allows rapid measurements with minimal or no sample preparation.

**Conceptual note**: Total N content does not necessarily reflect plant N status. While reference methods quantify total N pools, plant N status is a physiological construct integrating N allocation, remobilization, and metabolic use efficiency. Accordingly, indirect optical and spectral approaches primarily capture physiological consequences of N availability (e.g., chlorophyll content, photosynthetic performance, canopy structure), rather than N content itself.

In addition to the above-mentioned approaches, BIS has recently gained increasing attention. BIS is based on measuring the electrical resistance and capacitance of plant tissues (Prasad and Roy 2020). Since these parameters are sensitive to cell structure, water content, and ion concentration (Ando et al. 2014a, b; Jinyang et al. 2016; Meiqing et al. 2016; Li et al. 2017; Serrano-Finetti et al. 2023; Kovács et al. 2025e), BIS may be directly applicable for detecting leaf N concentration (Muñoz-Huerta et al. 2014; Li et al. 2017; Basak et al. 2020). The method is fast, non-destructive, and highly compatible with optical techniques. However, the number of scientific studies on BIS for nutrient detection, particularly for N, remains limited.

## Principles and applications of bioimpedance spectroscopy in plant physiological research

Bioelectrical impedance ($Z$, Ω) is a complex physical quantity defined as the ratio of voltage to current, describing the electrical resistive and capacitive properties of biological tissues (Jinyang et al. 2016; Meiqing et al. 2016; Li et al. 2017). Under alternating current (AC) conditions, both voltage and current vary sinusoidally over time, as expressed by the following relationship (Liu et al. 2021):

1 $$ U\left( t \right) = U_{0} \sin \left( {\omega t} \right) $$

2 $$ I\left( t \right) = I_{0} \sin \left( {\omega t + \phi } \right) $$

where $\omega =2\pi f$ is the angular frequency, f is the frequency (Hz), t is time, and $\phi$ is the phase angle, representing the phase shift between voltage and current.

From this relationship, impedance can be expressed in complex form as:

3 $$Z\left(\omega \right)=\frac{U\left(\omega \right)}{I\left(\omega \right)}=R\left(\omega \right)+jX\left(\omega \right)$$

where $R=\left|Z\right|\mathrm{cos} \left(\phi \right)$ is the ohmic (resistive) component, $X=\left|Z\right|\mathrm{sin} \left(\phi \right)$ is the capacitive reactance, and *j* is the imaginary number.

**Fig. 1:**
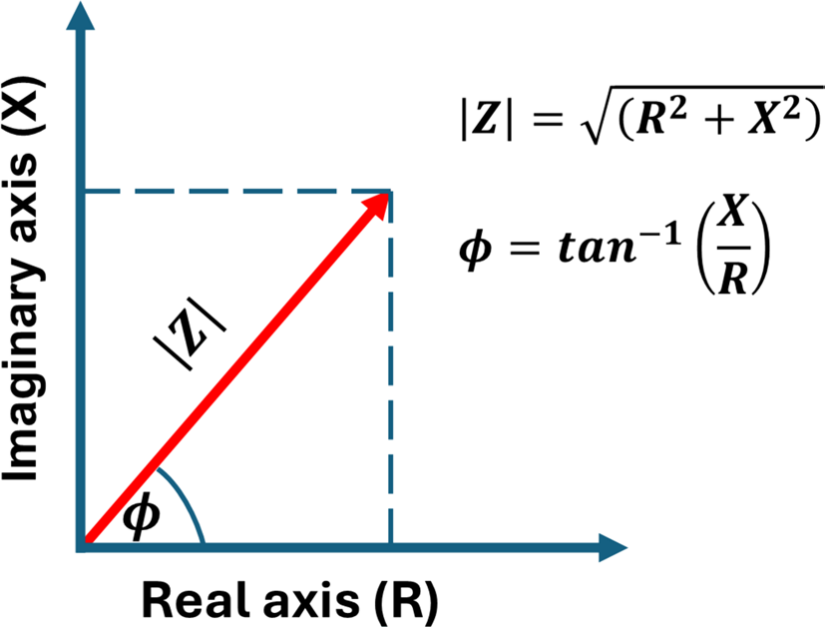
Vector diagram of complex impedance ($Z$ – magnitude or absolute value of impedance; $R$ – real part of the impedance; $X$ – imaginary part of the imepedance; $\phi$ – phase angle)

The magnitude of the impedance can be calculated from the quadratic sum of the resistive and capacitive components as follows (Fig. 1):

4 $$\left|Z\right|=\sqrt{\left({R}^{2}+{X}^{2}\right)}$$

This quantity represents the total electrical resistance of the tissue to alternating current, and its frequency dependence provides insights into the ionic conductivity and capacitive properties of the tissue (Liu et al. 2021).

**Fig. 2:**
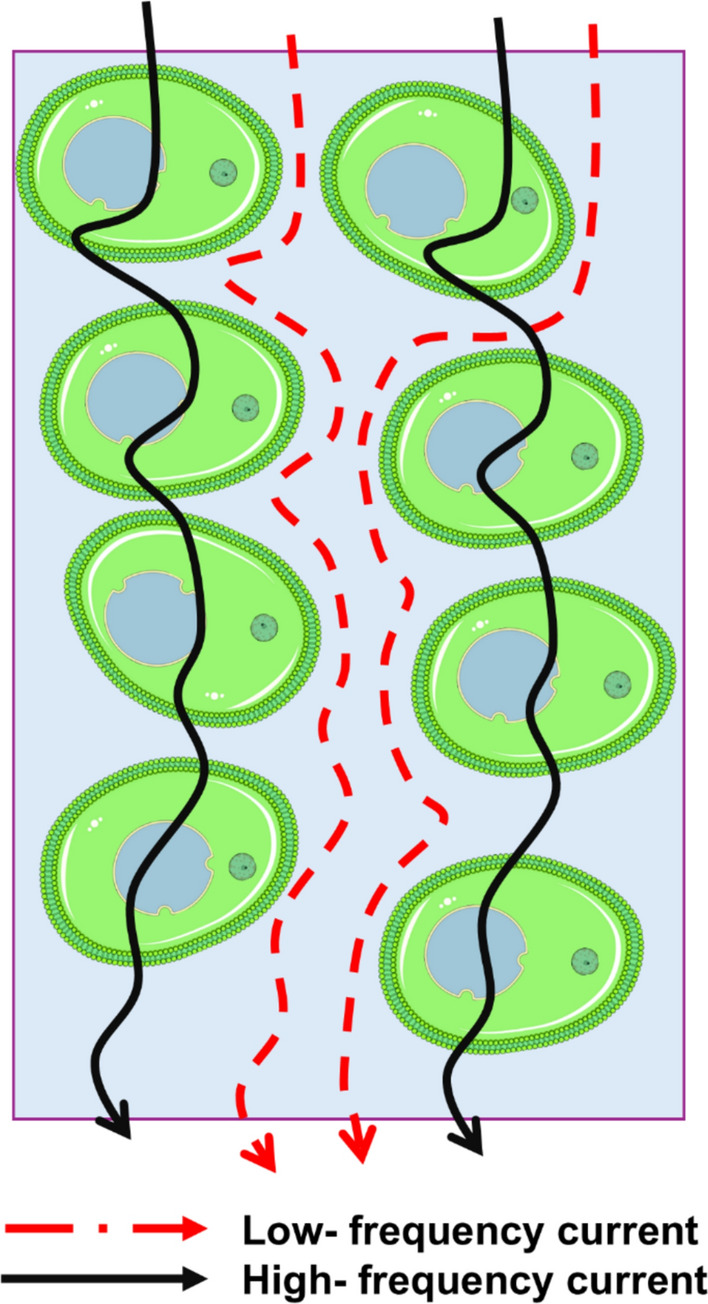
Schematic illustration of how low- and high-frequency electrical currents pass through plant tissue

The electrical conductivity of plant tissues is primarily determined by the presence and mobility of ions (Prasad and Roy 2020). The electric current within the tissue propagates through two main pathways: the apoplast (the region formed by cell walls and extracellular spaces) and the symplast (the intracellular space enclosed by the plasma membrane) (Fig. 2). Both compartments exhibit ohmic resistance; however, the plasma membrane separating them has unique electrical properties; it becomes polarized under an electric field and behaves as a capacitor (Zhou et al. 2025).

Thus, the electrical properties of plant tissues are determined by their cellular components and structural organization. When two tissue structures differ, for example, in cell wall thickness, water content, or ion distribution, these differences are reflected in distinct patterns within the impedance spectrum (Zhao et al. 2019; Serrano-Finetti et al. 2023).

The electrical impedance of biological tissues is a complex quantity composed of a real (resistive) and an imaginary (capacitive) component. These components are determined by the electrical resistance and capacitance of different structural elements within the tissue, such as conductive solutions, membranes, and cell walls (Jócsák et al. 2019). From an electrical perspective, the cell membrane acts like a capacitor with relatively high resistance to current flow, and its capacitance is frequency-dependent (Zhang et al. 1992, 2024).

At low frequencies, the alternating current primarily passes through the apoplastic (extracellular) fluid space, because the high capacitance of the membrane prevents current from entering the cell interior. In this case, the membrane behaves almost like an insulator, and the ionic medium inside the cells (the cytoplasm) is largely excluded from conduction (Jamaludin et al. 2015; Zhao et al. 2019; Jócsák et al. 2019; Basak et al. 2020). With increasing frequency, the capacitive reactance of the membrane decreases, its resistance becomes nearly negligible at high frequencies, and the current then flows through both the apoplast and symplast, resulting in lower total impedance (Jinyang et al. 2016; Meiqing et al. 2016; Li et al. 2017; Zhao et al. 2019).

The impedance spectrum of living tissues can be interpreted over a wide frequency range (1 Hz–10^12^ Hz), containing four characteristic dispersion regions (Grimnes and Martinsen 2005):

i) α-dispersion (1 Hz–a few kHz): associated with counter-ion movement along membrane surfaces, active membrane effects, ion channel activity, and diffusion processes (Nouaze et al. 2022).
ii) β-dispersion (10 Hz–1 MHz): reflects the polarizability and integrity of cell membranes; this is the most diagnostic range in BIS measurements of plant tissues (Grimnes and Martinsen 2005).
iii) γ- and δ-dispersion (MHz–GHz range): related mainly to molecular dipole relaxation and the dielectric response of water molecules, these ranges are rarely used in studies of living plant tissues (Bart et al. 2005).

Among these ranges, the β-dispersion range provides the most physiological information about plant tissues, reflecting the condition of the cell membrane, the water content, and the ion distribution, all essential for identifying stress responses, nutrient deficiency, and membrane injury (Ando et al. 2014a, b; Jinyang et al. 2016; Li et al. 2017; Meiqing et al. 2016; Serrano-Finetti et al. 2023).

The impedance of living plant tissues generally shows a decreasing trend with increasing frequency, while the phase angle varies across different frequency ranges. This behavior indicates that certain cellular components, such as membrane structures and macromolecular systems, cannot follow the changes in the electric field at all frequencies – meaning that their polarization rate is limited (Zhang and Willison 1993; Koklu et al. 2016; Zhang et al. 2022b).

In the α-range, the limiting factor is the diffusion and mobility of ions; in the β-range, it is the polarizability of membranes; and in the γ- and δ-ranges, the limitation arises from the loss of dipolar relaxation in water molecules and macromolecules (Prasad and Roy 2020).

The frequency dependence of impedance thus reflects the dynamics of cell-level electrical responses. At low frequencies, the capacitive behavior of the membrane determines the current pathway, while at higher frequencies, cytoplasmic conductivity becomes dominant. Typically, the electrical resistance of the intracellular fluid space is about one order of magnitude lower than that of the apoplast, whereas the membrane resistance is roughly one order of magnitude higher (Grimnes and Martinsen 2005).

The measured impedance spectrum does not only reflect the interior properties of the tissue, but also includes contributions from the electrodes and the damaged tissue regions between them. This occurs because the needle or flat electrodes used for measurement often cause cellular injury upon tissue penetration. The effects of contact impedance and electrode polarization become particularly significant at low frequencies. These issues can be mitigated by selecting suitable electrode materials and geometries (e.g., Ag/AgCl electrodes, optimized electrode spacing) and by applying a four-electrode measurement configuration (Zhang et al. 1992; Zhang and Willison 1992; Grimnes and Martinsen 2005; Serrano-Finetti et al. 2023).

## Equivalent circuit models of bioimpedance and their physiological interpretation

Several cell modeling approaches have been developed to describe the electrical behavior of biological tissues. The Cole–Cole model (Cole 1932) was the first to describe the impedance–frequency relationship as an arc-shaped curve, illustrating the dispersive nature of the electrical response. For plant tissues, this approach was further developed by Hayden et al. (1969), who proposed a model based on the analogy of a cell-level electrical structure.

The Hayden model (Hayden et al. 1969) describes the electrical properties of plant cells using three main components: extracellular fluid resistance (${R}_{1}$), intracellular fluid resistance (${R}_{2}$), and the capacitance (${C}_{m}$) and resistance (${R}_{m}$) of the cell membrane, which together form the basis of the system. In practice, the ${R}_{m}$ is several orders of magnitude higher than the other components, and thus it is often neglected (Jinyang et al. 2016; Meiqing et al. 2016; Li et al. 2017; Prasad and Roy 2020), allowing the model to be applied in a simplified form (Wu et al. 2008). This simplified Hayden model represents the electrical structure of a single cell and can be illustrated on a Cole–Cole (Nyquist) plot as an ideal semicircular curve.

Over time, several advanced equivalent circuit models have been developed to describe the electrical behavior of plant tissues under different physiological and stress conditions. These include the Double-Shell (DBS) model (Zhang and Willison 1992; Kovács et al. 2025e), the Randles model (Hamed et al. 2024), and the Gratus Citrus model (Juansah et al. 2012). Each model reflects a different level of structural and functional complexity. While the Cole–Cole and Hayden models provide a general description of single-cell or tissue-level responses, the DBS model accounts for both intracellular and vacuole cell compartments, and Randles-type circuits are mainly used to represent electrochemical processes occurring at interfaces. These models have been successfully applied to characterize plant responses—such as in tomato, eggplant, kiwi, and citrus species—to drought, metal toxicity, and freezing stress.

In the above-mentioned models, the CPE (Constant Phase Element) is increasingly applied to describe the non-ideal capacitive behavior of cell membranes. This approach provides a more realistic representation of plant tissues, as it accounts for structural heterogeneity, non-uniform charge distribution, and variability in ion mobility (Ando et al. 2014a, b; Jinyang et al. 2016; Meiqing et al. 2016; Li et al. 2017; Serrano-Finetti et al. 2023; Kovács et al. 2025c, e).

The CPE is characterized by the parameters Q and the fractional exponents α or β, which can take values between 0 and 1. These exponents quantify the deviation of the system from ideal capacitive behavior. When α or β equals 1, the membrane behaves as an ideal capacitor, whereas values approaching 0 indicate a strongly dispersive and non-ideal response, typical of structurally heterogeneous or stressed biological tissues (López-Villanueva and Rodríguez Bolívar 2022; Kovács et al. 2025b, e).

It is important to note that the CPE parameters ($Q$, α, β) are not only mathematical elements that improve curve fitting. The α and β exponents reflect the degree of membrane integrity and heterogeneity: values close to unity are associated with intact and homogeneous membranes, while decreasing values suggest stress-induced membrane disorganization, increased leakage, or structural irregularities. Consequently, variations in α and β can be interpreted as early indicators of physiological stress and tissue heterogeneity in plant systems (Prasad and Roy 2020).

Through these models, electrical bioimpedance can be interpreted not only as a physical parameter but also as a physiological indicator, as it sensitively reflects membrane integrity, water potential, nutrient status, and stress responses in plant tissues (Ando et al. 2014a, b; Jinyang et al. 2016; Li et al. 2017; Meiqing et al. 2016; Serrano-Finetti et al. 2023).

If the goal is to relate N metabolism with different cell models, the DBS model appears to be the most suitable choice (Kovács et al. 2025d). The DBS model was first applied to plant (lettuce) tissues by Zhang et al. (1992). It describes the cell as having two distinct boundaries—the plasma membrane and the tonoplast, the vacuole membrane that separates the cytosol from the vacuole compartment and plays a key role in intracellular ion storage and compartmentalization.

**Fig. 3:**
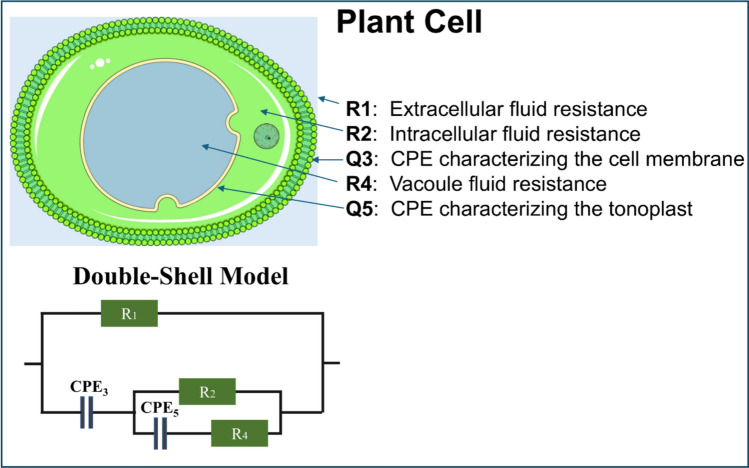
Equivalent electrical circuit of the Double-Shell (DBS) model and the simplified structural representation of a plant leaf based on the DBS parameters (Kovács et al. 2025a)

The DBS model includes the resistance of the extracellular fluid resistance (${R}_{1}$), the intracellular fluid resistance (${R}_{2}$), and the vacuole parameters (${R}_{4}$ and ${Q}_{5}$). Here, ${R}_{4}$ represents the resistance of the vacuole, while ${Q}_{5}$ corresponds to the constant phase element (CPE) describing the tonoplast (vacuole membrane). In addition, ${Q}_{3,}$ represents another CPE element that characterizes the capacitive behavior of the plasma membrane (Fig. 3).

The parameters ${R}_{4}$ and ${Q}_{5}$ therefore provide direct information about the ionic composition of the vacuole and the functional state of the tonoplast. Since the vacuole serves as a major N pool, these elements are physiologically linked to N storage and its remobilization under stress or nutrient deficiency conditions (Kovács et al. 2025d).

The total impedance of the DBS model, $Z\left(j\omega \right),$ can be expressed as follows:

$$\begin{array}{ccc}Z\left(j\omega \right)=\frac{{R}_{1}\left(\frac{1}{{\left(j\omega \right)}^{\beta }{Q}_{3}}+{Z}_{partial}\right)}{{R}_{1}+\frac{1}{{\left(j\omega \right)}^{\beta }{Q}_{3}}+{Z}_{partial}},& \mathrm{ahol}& {Z}_{partial}=\frac{{R}_{2}\left({R}_{4}+\frac{1}{{\left(j\omega \right)}^{\alpha }{Q}_{5}}\right)}{{R}_{2}+{R}_{4}+\frac{1}{{\left(j\omega \right)}^{\alpha }{Q}_{5}}}\end{array}$$. (5).

In Eq. (5), $\omega$ denotes the angular frequency, and $j=\sqrt{-1}$​ represents the imaginary unit. The parameters $\alpha \in (\mathrm{0,1}]$ and $\beta \in (\mathrm{0,1}]$ describe the CPE (Constant Phase Element) behavior.

The impedance of the $CP{E}_{5}$ element is given by ${Z}_{CP{E}_{5}}=1/\left({\left(j\omega \right)}^{\alpha }{Q}_{5}\right),$ which defines the CPE-based impedance of the ${Q}_{5}$ capacitor. Similarly, the impedance of the $CP{E}_{3}$ element is expressed as ${Z}_{CP{E}_{3}}=1/({\left(j\omega \right)}^{\beta }{Q}_{3})$, representing the CPE-based relaxation behavior of the ${Q}_{3}$ capacitor. The parameters α and β provide greater flexibility in describing the impedance spectrum, as they allow for modeling non-ideal capacitive membranes that separate the different fluid compartments (Odry et al. 2021).

The time constants corresponding to the plasma membrane capacitance (${C}_{m}$) and tonoplast capacitance (${C}_{t}$) can be calculated as ${\tau }_{1}$ and ${\tau }_{2}$ respectively (Ando et al.; Jinyang et al. 2016; Meiqing et al. 2016; Serrano-Finetti et al. 2023; Kovács et al. 2025b, e, d).

## Limitations and challenges of bioimpedance applications in nutrient uptake and N status assessment in plants

BIS was applied relatively early to study the water content of plant roots and stems, for example in potato and alfalfa (Hayden et al. 1969). Barbosa et al. (2022) reported a strong correlation between leaf water content and the absolute value of impedance in their two-electrode measurements. However, due to the limitations of the two-electrode technique, it was not possible to accurately determine individual components of the tissue’s electrical model, such as membrane capacitance or intracellular fluid resistance.

Regarding measurement setup, this limitation comes from the contact impedance of the electrodes, which can interfere with the measurements even when using non-polarizable electrodes (e.g., Ag/AgCl). To overcome this issue, the four-electrode configuration has become widely used, as it minimizes the influence of the electrodes on the measurement, thereby providing more accurate data on the electrical properties of plant tissues. Furthermore, this configuration allows the use of polarizable or capacitive electrode materials, thus improving the flexibility and reproducibility of in vivo measurements (Morales et al. 2021; Serrano-Finetti et al. 2023; Kovács et al. 2025b, d, e).

Environmental stress factors and their physiological consequences have been a central focus of plant physiology research for decades. Classical methods, such as the determination of chlorophyll content (Arnon 1949), protein concentration, or stress-induced enzyme activity, provide accurate results but are destructive techniques. Consequently, they are unsuitable for repeated measurements on the same individuals, making it difficult to monitor the temporal dynamics of stress responses (Jócsák et al. 2019). One of the main objectives of modern plant physiology is the development of non-destructive, in vivo and in situ measurement techniques capable of continuously monitoring plant stress responses (Zhou et al. 2024).

Early studies demonstrated that BIS is highly sensitive to changes in cell membrane integrity, water status, and the electrical properties of cellular compartments. For instance, in potato and apple, cooling caused an increase in both intracellular and extracellular resistance (Hayden et al. 1969; Toyoda and Tsenkova 1998), while during drying, resistance values changed markedly in parallel with decreasing water content (Vozáry et al. 1999).

In cold stress studies, BIS parameters—particularly intracellular fluid resistance—showed strong correlations with freezing resistance capacity (Hietala et al. 1998; Väinölä and Repo 2000). BIS has also proven to be a valuable tool for assessing environmental stress effects on plants. For example, radiation and heavy metal exposure were shown to alter the electrical resistance of plant tissues (Felföldi et al. 1993). Under nickel stress, a slight increase in resistance was observed in guava plants, likely due to the accumulation of Ni^2+^ ions in the extracellular fluid space (Bazihizina et al. 2015).

Moreover, BIS can detect subtle changes in root and soil interactions induced by mycorrhizal symbiosis or cold treatment, even when other methods lack sufficient sensitivity (Repo et al. 2014). The technique can also be used to monitor virus-induced cell necrosis, such as that caused by the tomato mosaic virus (Greenham et al. 1972). Jócsák et al. (2010) demonstrated that in pea roots, changes in impedance under flooding and cadmium stress accurately reflected alterations in cellular structure.

Although the use of BIS is becoming increasingly widespread in plant research, most studies have so far focused primarily on water deficit and general plant health status (Garlando et al. 2022; Jamaludin et al. 2015; Reynolds et al. 2023; Serrano-Finetti et al. 2023; Wang et al., 2020b; Zhang et al. 2024, 2022a, b), while research on nutrient-specific effects has remained limited.

Meiqing et al. (2016) demonstrated that in phosphorus-deficient tomato plants (*Solanum lycopersicum* L.), extracellular fluid resistance increased, while cell membrane capacitance decreased significantly, reflecting a decline in the ion conductivity of root and leaf tissues. Similarly, Jinyang et al. (2016) reported that potassium deficiency led to a marked increase in intracellular resistance and a decrease in membrane capacitance, indicating structural damage to the cell membrane. BIS measurements showed a strong correlation with plant K^+^ content, suggesting its suitability for the early detection of nutrient deficiency and for monitoring membrane-related physiological changes.

Nevertheless, growing evidence suggests that BIS may be capable of detecting nutrient deficiencies, particularly N deficiency (Muñoz-Huerta et al. 2014; Li et al. 2017; Basak et al. 2020; Kovács et al. 2025b, d). Previous studies have also shown that the method can be used to identify deficiencies in phosphorus (P) (Meiqing et al. 2016) and potassium (K) (Jinyang et al. 2016). A more recent study confirmed that BIS can also be used to detect iron deficiency (Hamed et al. 2023). The researchers found that in Fe-deficient plants, both intracellular and extracellular fluid resistance increased, while cell membrane capacitance decreased, signaling a loss of membrane integrity. These results confirmed that BIS is a sensitive, non-destructive tool for detecting micronutrient deficiencies, particularly by tracking early alterations in cell membrane function and ion conductivity, making it suitable for diagnosing iron stress in plants.

Table 2 summarizes the number of BIS studies on nutrient diagnostics published between 2012 and 2025. N is the most studied nutrient, while P, K, and Fe are much less researched. The increasing number of N-related studies highlights the growing interest in developing non-destructive BIS-based methods for monitoring nutrient status in plants.

**Table 2:** Temporal distribution of bioimpedance spectroscopy (BIS) studies focusing on plant nutrient diagnostics (2012–2025)

Year	Species	Nutrient	Equivalent circuit model	BIS parameter(s)	Tissue	Reference
2012	Tomato	Mineral nutrient deficiency (N, P)	Not specified	IMV, Nutrition Index P(k) (EIS)	Stem	Tomkiewicz and Piskier (2012)
2014	Lettuce	N	Not specified	φmin, fmin, Zmin	Leaf	Muñoz-Huerta et al. (2014)
2016	Tomato	P	Modified model (CPE-based)	${R}_{\mathrm{e}}$, ${R}_{\mathrm{i}}$, ${C}_{m}$ (CPE-based EIS)	Leaf	Meiqing et al. (2016)
2016	Tomato	K	Modified model (CPE-based)	${R}_{\mathrm{e}}$, ${R}_{\mathrm{i}}$, ${C}_{m}$ (CPE-based EIS)	Leaf	Jinyang et al. (2016)
2017	Tomato	N	Modified model (CPE-based)	${R}_{\mathrm{e}}$, ${R}_{\mathrm{i}}$, ${C}_{m}$ (CPE-based EIS)	Leaf	Li et al. (2017)
2020	Canola, wheat, soybean, corn	N	Not specified	|Z|	Leaf	Basak et al. (2020)
2023	Tomato	Fe	Single-dispersion Cole model (CPE-based)	|Z| (10 kHz), ${R}_{s}$, ${R}_{p}$, CPE-T, CPE-P	Stem	Hamed et al. (2023)
2024	Tomato	Fe	Single-dispersion Cole model (CPE-based)	Z| (10 kHz), ${R}_{s}$, ${R}_{p}$, CPE-T, CPE-P	Stem	Altana et al. (2024)
2025	Cucumber	N	Double-Shell model with CPEs	${R}_{1}$, ${R}_{2}$, ${R}_{4}$, ${C}_{m}$, ${C}_{t}$, α, β	Leaf	Kovács et al. (2025c)
2025	Lettuce	N	Double-Shell model with CPEs	${R}_{1}$, ${R}_{2}$, ${R}_{4}$, ${C}_{m}$, ${C}_{t}$, α, β	Leaf	Kovács et al. (2025d)
2025	Cucumber	N	Double-Shell model with CPEs	${R}_{1}$, ${R}_{2}$, ${R}_{4}$, ${C}_{m}$, ${C}_{t}$, α, β	Leaf	Kovács et al. (2025a)

${R}_{1}$/${R}_{\mathrm{e}}$ and ${R}_{2}$/${R}_{\mathrm{i}}$ denote the resistances of the extracellular (apoplast) and intracellular (symplast/cytosol) spaces, respectively; ${R}_{4}$ represents vacuole fluid resistance and ${C}_{t}$ the tonoplast capacitance, both defined only in Double-Shell models, whereas ${C}_{m}$ describes plasma membrane capacitance; ${R}_{s}$ and ${R}_{p}$ are series and parallel resistances in Cole-type models, CPE-T and CPE-P describe tissue-level and polarization-related non-ideal capacitive behavior; |Z| is the impedance magnitude at the indicated frequency; and IMV and Nutrition Index P(k) are empirical, model-independent impedance indices, α and β are fractional exponents of constant phase elements associated with the tonoplast and plasma membrane, respectively; and $\phi$ min, f min, and Z min refer to the phase minimum, its corresponding frequency, and the impedance magnitude at that frequency

In summary, although several studies have investigated the relationship between BIS and plant nutrient status, particularly N deficiency, the number of such studies remains limited, and the available literature data are still relatively scarce in this field. To further illustrate how BIS reflects nutrient-related physiological changes, several case studies focusing on N deficiency are summarized below.

Li et al. (2017) demonstrated that N deficiency significantly affected the bioelectrical properties of plant cells, particularly the extracellular and intracellular fluid resistance and the cell membrane capacitance. In their experiments, the impedance of tomato leaves was measured over a frequency range of 1 Hz–1 MHz, and the data were analyzed using a modified Cole–Cole equivalent circuit model, which included elements representing the extracellular and intracellular fluid resistances and a constant phase element (CPE) for the cell membrane. The results showed that the total impedance magnitude was significantly higher in N-deficient plants compared to those supplied with adequate N. This increase was attributed to the reduced ion concentration in the leaves, which led to a decrease in electrical conductivity. The impedance curves showed that the effects of different N levels appeared mainly in the low-frequency range (1 Hz–10 kHz), where the electric current passes through the extracellular fluid space since the cell membrane capacitance still blocks current flow into the intracellular compartment. At higher frequencies, the differences decreased because the capacitive reactance of the membrane decreased, allowing current to pass through the cells. This behavior reflects the β-dispersion phenomenon characteristic of biological tissues. Under N deficiency, the cell membrane capacitance dropped sharply below 2.69% N content, indicating cell membrane damage and loss of structural integrity. Meanwhile, extracellular fluid resistance decreased while intracellular fluid resistance increased, suggesting ion leakage from the cells into the extracellular fluid space. Consequently, the extracellular/intracellular resistance ratio approached unity, serving as a sensitive indicator of membrane injury. The study further showed that there was a positive correlation between cell membrane capacitance and leaf N content: as N increased, so did the cell membrane capacitance, indicating improved membrane elasticity and ion conductivity. In contrast, N deficiency led to increased cell membrane permeability, ion imbalance, and substantial changes in the electrophysiological behavior of cells. The most sensitive frequency range was identified between 2.51 kHz and 13.59 kHz, from which a regression model was developed to estimate leaf N content with a high correlation coefficient (R^2^ = 0.86). In this study, the use of a four-electrode configuration minimized electrode polarization and contact impedance effects, thereby improving the reliability of the measured impedance spectra. Nevertheless, changes in extracellular fluid resistance may also reflect variations in the NO_3_⁻ concentration within the extracellular fluid space, which could directly influence the BIS readings (Crawford and Glass 1998; Nikolic et al. 2012; Hu et al. 2014).

### .

Basak et al. (2020) developed a portable BIS measurement system based on the AD5933 chip to estimate the N status of different crop species (maize, wheat, soybean, and canola) under *vivo* conditions. Measurements conducted within the 5–15 kHz frequency range revealed that N-deficient plants exhibited significantly higher impedance values, whereas increasing N supply resulted in a decrease in impedance, indicating improved ionic conductivity within plant tissues. The authors statistically confirmed the differences between N treatments, and multivariate regression models showed a strong correlation between leaf N concentration and BIS parameters. They also identified the optimal frequency range for detecting N status to be between 8.7 and 12 kHz, where BIS responded most sensitively to variations in plant N supply. In this study, BIS measurements were performed using a two-electrode configuration with surface ECG electrodes, which, while suitable for portable and rapid field measurements, may be more sensitive to electrode tissue contact impedance. As a result, part of the observed impedance variation, particularly in the low frequency range, may include contributions from the electrode interface in addition to plant tissue properties.

Muñoz-Huerta et al., (2014) applied BIS to assess the N status of lettuce (*Lactuca sativa* L.). They investigated the relationship between plant N content and the minimum phase angle of the impedance spectrum. In their experiment, four groups of plants were treated with nutrient solutions containing different NO_3_⁻ concentrations, and measurements were conducted in the 1–100 kHz frequency range using a non-invasive electrode setup. The authors analyzed the minimum phase angle ($\phi$ min) and the corresponding frequency (f min) and found that f min showed a strong positive correlation with total plant nitrogen content (R^2^ = 0.99). In contrast, the minimum phase ($\phi$ min) and minimum impedance (Z min) values exhibited only weak or negative correlations. Increasing frequency reflected a higher ion concentration and conductivity in plant tissues, as NO_3_⁻ ions reduced extracellular fluid resistance and shortened the relaxation time. In this study, BIS measurements were performed using a two-electrode configuration with stainless steel needle electrodes inserted into the leaf midrib. While this approach enabled noninvasive measurements, electrode tissue contact impedance and local tissue disturbance may have contributed to the measured phase response. Moreover, because the analysis relied on global spectral features without equivalent circuit modeling, the physiological attribution of $\phi$ min and f min remains indirect and integrates multiple tissue and interface related effects.

Kovács et al. (2025c) demonstrated that BIS effectively reflects N-related physiological changes in lettuce genotypes. Across five N treatments, extracellular fluid resistance showed strong negative correlations with total N and NO_3_⁻ content, while cell membrane capacitance correlated positively with membrane stability. The study also revealed genotype-dependent responses, indicating that BIS sensitively captures N uptake dynamics and cell membrane integrity, providing a non-destructive tool for monitoring plant B status. More recent work by Kovács et al. (2025a) further confirmed the applicability of BIS for assessing N status in plants. Their study on cucumber demonstrated that low N supply markedly increased extracellular fluid resistance and decreased membrane capacitance, reflecting reduced NO_3_⁻ availability and impaired membrane integrity. More recently, a follow-up study extended these findings by demonstrating that BIS, combined with Double Shell modeling and particle swarm optimization, can detect early N deficiency and reliably classify plant N status in cucumber genotypes, while revealing genotype-dependent differences in vacuole fluid responses (Kovács et al. 2025a). In these studies (Kovács et al. 2025b, d, a), BIS measurements were performed using a four-electrode configuration combined with equivalent circuit modeling, which minimized electrode polarization and contact impedance effects and allowed a more direct physiological interpretation of extracellular and membrane-related impedance parameters.

Although BIS appears to be a promising technique for evaluating the N status of plants, the number of studies remains limited. In most cases, electrical equivalent circuit models were not applied to interpret the impedance spectra (Muñoz-Huerta et al. 2014; Basak et al. 2020). Table 3 summarizes the available literature on BIS-based studies assessing N deficiency in plants, highlighting both the consistent trends and the genotype-dependent variations observed in the key electrical parameters.

**Table 3:** Overview of BIS-based studies evaluating N deficiency in plants based on the current literature

Parameter group	Dominant frequency range	Observed trend under low N supply	Biological interpretation	Representative studies
Extracellular fluid resistance	Low-frequency range (≈1 Hz–10 kHz)	Generally ↑; ↓ reported in (Li et al. 2017)	Generally, increases under low nutrient supply due to reduced extracellular ion conductivity	(Muñoz-Huerta et al. 2014; Basak et al. 2020; Kovács et al. 2025c, d)
Intracellular fluid resistance	Upper low to mid-frequency range (≈1–50 kHz)	**↑** in all reported studies	Lower intracellular conductivity and increased ionic resistance due to nutrient depletion and osmotic imbalance	(Li et al. 2017; Kovács et al. 2025b, d)
Vacuole fluid resistance	Upper mid-frequency range (≈30–100 kHz)	**↑** where available	Depletion of the vacuole as an ionic storage pool; however, this response showed strong genotype-dependent variation between the two lettuce genotypes	(Kovács et al. 2025c, d)
Cell membrane capacitance	β-dispersion range (≈2–50 kHz)	**↓** in all reported studies	Decreased charge storage capacity and impaired membrane fluidity and structural integrity under nutrient deficiency	(Li et al. 2017; Kovács et al. 2025b, d)
Tonoplast capacitance	Upper mid-frequency range (≈30–100 kHz)	**↓** where available	Reduced tonoplast elasticity and vacuole charge storage capacity	(Kovács et al. 2025c, d)

Note: the direction and magnitude of change for some parameters were genotype-dependent. The arrows show how the parameters changed under low N supply: ↑ means an increase, ↓ means a decrease;

## Concluding remarks and future perspectives

BIS is emerging as a promising, non-destructive method for assessing the N status of plants. Studies consistently show that N deficiency alters key electrical parameters of plant tissues: extracellular and intracellular fluid resistances change, while cell membrane capacitance decreases, reflecting reduced ion conductivity, loss of membrane integrity, and changes in vacuole storage. These electrical variations can be detected in vivo within defined frequency ranges and, in some cases, correlate strongly with leaf N content. However, in most studies, BIS parameters were correlated with total N content, which does not represent the inorganic N forms. Therefore, future research should also include the evaluation of changes in NO_3_⁻ content.

Since current evidence remains limited, most available studies have evaluated only a few genotypes and a few number of nutrient treatments, often under controlled conditions, and in many cases without applying a complete equivalent circuit model. In addition, BIS has been used mainly to describe general stress responses (such as drought, freezing, and heavy metal exposure) and water status, whereas nutrient-specific interpretations are less developed. Consequently, BIS is not yet a routine tool for nutrient diagnostics in agronomy.

A central unresolved issue is the distinction between N deficiency and water deficit. Both stresses induce clear and sometimes similar changes in extracellular and intracellular resistance, as well as in membrane-related parameters. While BIS is already well established for monitoring water status, water deficit itself strongly affects the same BIS parameters that are now being proposed as N indicators. This means that an increase in extracellular fluid resistance or a decrease in membrane capacitance cannot automatically be interpreted as N deficiency unless the water status is known simultaneously. Distinguishing these two signals remains one of the greatest practical challenges and a major physiological limitation for the use of BIS as an in vivo N sensor.

Another critical limitation is that most N-related BIS studies quantified N status only as total N (typically using the Kjeldahl method) and did not determine the actual ionic forms present in the tissues. In particular, the relationship between extracellular NO_3_⁻ concentration and extracellular fluid resistance has rarely been measured directly (Kovács et al. 2025d), and often only inferred indirectly. This represents a key weakness because NO_3_⁻ in the apoplast is mobile, actively exchanged between apoplast and symplast, and regulated by light, pH, and H⁺–ATPase activity. These dynamics directly influence charge transport and thus the electrical resistance measured in the extracellular fluid space. Without determining the amount of apoplastic NO_3_⁻, it is not yet possible to confirm whether the observed BIS changes are caused by NO_3_⁻ depletion, altered membrane function, or simply by reduced tissue hydration (Kovács et al. 2025c).

Given the complexity and multivariate characteristics of BIS datasets, future progress will likely require the integration of large-scale phenotypic, physiological, and BIS measurements collected across genotypes, phenological stages, and nutritional regimes. Such comprehensive datasets could provide a basis for applying artificial intelligence and machine learning approaches to identify patterns, classify stress states, and improve the biological interpretation of BIS signals.

Based on the current evidence, several research priorities can be identified:

(i) Future studies should include direct measurements of extracellular NO_3_⁻ and NH_4_⁺, parallel with BIS data from the same leaves. Combining BIS with apoplastic infiltration and NO_3_⁻ quantification would clarify whether changes in extracellular fluid resistance truly reflect NO_3_⁻ availability or water status.
(ii) Experiments should combine different levels of N supply with both well-watered and drought conditions to separate N and water effects. This design would help identify BIS parameters that respond specifically to N deficiency rather than dehydration.
(iii) Advanced equivalent circuit models, such as the Double-Shell (DBS) model, should be applied to interpret BIS data.
(iv) Beyond N, other nutrients such as P, K, and Fe should also be evaluated under controlled conditions to determine whether each nutrient produces a distinct electrical signature.
(v) Statistical and classification models should be developed to distinguish N deficiency from drought effects using multiple BIS parameters rather than a single resistance value.

In summary, BIS is technically sensitive to plant N status, but its physiological interpretation is still incomplete. The main challenges include linking extracellular fluid resistance to apoplastic NO_3_⁻, separating N and water effects, and integrating physiologically meaningful models. Addressing these issues will be essential for using BIS as a reliable, non-destructive diagnostic tool for N assessment under real growth conditions.

## Data Availability

Not Applicable.
